# Object Affordances Potentiate Responses but Do Not Guide Attentional Prioritization

**DOI:** 10.3389/fnint.2015.00074

**Published:** 2016-01-12

**Authors:** Yusuke Yamani, Atsunori Ariga, Yuki Yamada

**Affiliations:** ^1^Department of Psychology, Old Dominion UniversityNorfolk, VA, USA; ^2^Faculty of Psychology, Rissho UniversityTokyo, Japan; ^3^Faculty of Arts and Science, Kyushu UniversityFukuoka, Japan

**Keywords:** object affordances, visual search, stimulus-response compatibility, visual perception and attention, motor selection

## Abstract

Handled objects automatically activate afforded responses. The current experiment examined whether objects that afford a response are also prioritized for attentional processing in visual search. Targets were pictures of coffee cups with handles oriented either to the right or the left. Subjects searched for a target, a right-handled vs. left-handled coffee cup, among a varying number of distractor cups oriented in the opposite direction. Responses were faster when the direction of target handle and the key press were spatially matched than mismatched (stimulus-response compatibility (SRC) effect), but object affordance did not moderate slopes of the search functions, indicating the absence of attentional prioritization effect. These findings imply that handled objects prime afforded responses without influencing attentional prioritization.

Manual interaction with objects such as grabbing a cup of coffee requires accurate perception of objects and their spatial layout as well as sensorimotor integration. Gibson (1979/1986) defined possibilities for action that objects or environments offer as *object affordances*, and suggested that object affordances can potentiate specific actions. For example, a computer mouse affords reaching and holding, and a chair affords sitting.

Behavioral (de’Sperati and Stucchi, 1997; Craighero et al., 1998, 1999; Tucker and Ellis, 1998; Castiello, 1999; Ellis and Tucker, 2000; Creem and Proffitt, 2001; Phillips and Ward, 2002; Vainio et al., 2007; Linkenauger et al., 2009), electrophysiological (Goslin et al., 2012; Wilf et al., 2013), neuropsychological (Riddoch et al., 2003) and neuroimaging studies (Grèzes et al., 2003; Creem-Regehr et al., 2007) have demonstrated the automatic activation of afforded responses (but see Lindemann et al., 2006; Bub and Masson, 2010). Tucker and Ellis (1998), for example, asked subjects to view objects that afford either a right- or left-hand response and to make either a right- or left-hand key press to report whether each object was upright or inverted. Response times (RTs) were shorter when the responses were made by the afforded hand (compatible trials) than the non-afforded hand (incompatible trials), a form of the stimulus-response compatibility (SRC) effect (Proctor and Vu, 2006). In a similar study, action-relevant stimuli primed associated components of the afforded action such as grasp or wrist rotation (Ellis and Tucker, 2000), showing automatic extraction of action-relevant information from perceived objects.

More recent reports argued that the SRC effect in the previous paradigm conflated the affordance effect (e.g., Michaels, 1988; Tucker and Ellis, 1998) and the Simon effect (Simon, 1969), and demonstrated that the SRC effect can arise due to spatial mapping of the handle but not to a grasping affordance (Cho and Proctor, 2010; Pappas, 2014). Pappas (2014), for example, conducted a series of experiments manipulating object affordance information while retaining the SRC by presenting stimuli in a silhouette, and again showed the SRC effects. These results thus imply that object affordance by itself may not trigger automatic responses, but it remains unclear how much affordance information their subjects were able to extract from the silhouette stimuli and use for activating particular responses.

The literature thus suggests that visual objects can automatically activate afforded responses. Conversely, action preparation appears to modulate visual processing (Craighero et al., 1998, 1999) and attentional selection in visual search (Wykowska et al., 2009; Buttaccio and Hahn, 2011). For example, simple actions in a go/no-go task can affect subsequent visual search performance (Buttaccio and Hahn, 2011). Subjects in the study performed a go/no-go task where they responded (go) when a color name and a shape of the cued color matched and withheld their responses (no-go) when mismatched. Immediately after the go/no-go task, subjects performed a visual search task, finding a tilted line among vertical lines, where each item was presented with a colored shape. Their RTs in a visual search task were shorter when an array of search objects appeared within the primed object in the go/no-go task, showing that even simple actions to an object can influence speed of visual search.

What is the role of object affordance in attentional selection of graspable objects in a visual scene? Earlier work has demonstrated an attention cuing effect from objects with affordances (Tipper et al., 1998; Handy et al., 2003; Handy and Tipper, 2007; Garrido-Vásquez and Schubö, 2014). An event-related functional magnetic resonance imaging study (Garrido-Vásquez and Schubö, 2014), for example, demonstrated that visuospatial attention was allocated preferentially to affording objects, as shown by neural activation in not only the occipital lobe but also in dorsal regions of premotor and prefrontal cortices responsible for action planning based on visual information. The current study employed the *search asymmetry paradigm* to test whether in addition to priming an afforded response, such attention cuing effect translates into attentional prioritization in a visual search task.

In a standard visual search task, subjects are asked to make a speeded judgment of whether a search display contains a designated target object among some number of distractors, where the location of the target is *a priori* uncertain. The number of distractors within the display is varied, and RTs are analyzed as a linear function of display size (Wolfe, 1998). The slope of the RT × display size function is taken to represent search efficiency, and the intercept to reflect pre-search sensory processing and post-search response execution times. A search asymmetry exists between a pair of target and distractor items when one target-distractor mapping (e.g., Q as target vs. Os as distractors) produces more efficient search than the reversed mapping (e.g., O as target vs. Qs as distractors; Treisman and Souther, 1985; Treisman and Gormican, 1988; Wolfe, 2001; Yamani and McCarley, 2010). Often, the slope of the search function relating RTs to the number of distractors is indistinguishable from zero with the efficient mapping, indicating “pop-out” search, and greater than zero with the less-efficient mapping, indicating slow and effortful search (Wolfe, 1998). An asymmetry may obtain when the favored item in the target-distractor pair posses a distinctive feature, such as the line segment of a Q that is absent from an O (Treisman and Gelade, 1980). Alternatively, it may obtain when the favored item is an unfamiliar object (mirror-reversed objects; Malinowski and Hübner, 2001) and the disfavored item is familiar (canonically-oriented objects; Wolfe, 2001; Yamani and McCarley, 2011). This familiarity-driven search asymmetry effect could arise due to the difference in signal-to-noise ratio (SNR) between the two target-distractor mappings: a display containing the favored target item has a higher SNR because the unfavored distractor items requires more inefficient processing than the favored target item, but a display with the unfavored target item has a lower SNR (Rauschenberger and Yantis, 2006).

The present study employed the search asymmetry paradigm to examine whether object affordances not only potentiate a response automatically but also produce attentional prioritization in a visual search task. Note that the current experiment was not intended to examine the locus of the SRC effect (e.g., Cho and Proctor, 2010) but to test whether graspable objects attract visual spatial attention when they trigger automatic responses and modulates search efficiency. Stimuli were images of coffee cups with handles oriented to either the left or the right. We hypothesize that attentional cuing effect to an affording object translates to attentional prioritization of the object among others in a visual search task. It follows that the search slope for a right-handled cup target among left-handled cup distractors will be smaller when the subject responds to the right-handled target with the right hand than that for a left-handled cup target among right-handled cup distractors, and vice versa. In contrast, effects of automatic response potentiation are expected to manifest in search intercepts. Half of subjects responded to target-present trials with the index finger of the right hand and to target-absent trials with the index finger of the left hand, and this mapping was reversed for the other half. Thus, if the affordance of the cup handle automatically potentiates a response, search intercepts will be lower when the orientation of the handle matches the hand used for target-present responses (the SRC effect). If the predictions above are correct, the data should show two-way interactions of response mapping by target type on search intercepts and slopes.

## Materials and Methods

### Subjects

Seventy young adults (44 males, mean age = 19.6 years, mean laterality index = 0.65, *SD* = 0.34) were recruited from the community of the University of Illinois at Urbana-Champaign. All reported normal or corrected-to-normal vision. They received a course credit in exchange for participation.

### Apparatus

Stimuli were presented on a 19” CRT monitor with a refresh rate of 75 Hz and a resolution of 1280 × 1024 pixel. E-prime 2.0 (Psychology Software Tools, Pittsburgh, PA, USA) controlled presentation and timing of stimuli. Responses were made by mouse buttons. Subjects viewed the display from a distance of approximately 57 cm.

### Stimuli

**Figure 1:**
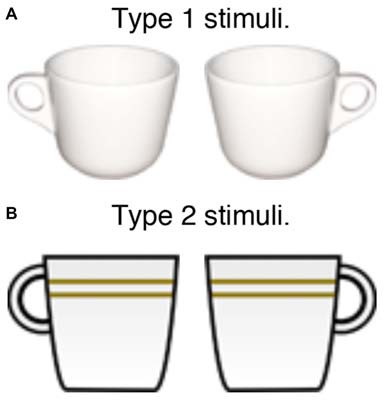
**Two sets of stimuli used for the experiment. (A)** Type 1 stimuli. **(B)** Type 2 stimuli.

The stimuli were displays of 4, 8, or 12 left-handled or right-handled coffee cups, a shaded and textured picture (type 1 stimuli: Figure 1A) for 28 subjects and a cartoon drawing (type 2 stimuli: Figure 1B) for the other 42 subjects to test whether the SRC effect arises only with realistic stimuli. Previous research (Pappas, 2014) indicates that stimuli carrying less affordance information can attenuate the SRC effect. Type 1 cups subtended 1.91° by 1.50°, and Type 2 cups subtended 1.91° by 1.63° respectively. Each item was positioned at 1 of 49 possible locations in a 7 × 7 imaginary grid, with a center-to-center distance of 2.45° between items, randomly jittered between 0° to 0.27° both horizontally and vertically. The stimuli were presented against a white background.

### Procedure

Prior to the experiment, all subjects filled out a modified version of the Edinburgh Handedness Inventory (Oldfield, 1971). A question regarding the daily use of a coffee cup was added to the inventory.

The subjects’ task was to search each display for the presence of a target cup oriented differently from the surrounding distractor cups, and to make a speeded response each trial indicating whether or not a target was present. Target-absent displays contained cups all oriented to the same direction. Target-present displays contained a single cup oriented in the opposite direction of the other cups. Half of trials were target-absent and the other half were target-present. Target orientation (left-handled vs. right-handled) alternated between blocks, with the order of blocks counterbalanced across subjects. Subjects were asked to rest their hands on either side of the mouse with index fingers on the buttons. Responses for target-present trials were executed with the index finger of one hand and those for target-absent trials were executed with the index finger of the other hand. Response mapping was counterbalanced across subjects.

Each trial began with a 400 ms blank screen, followed by the stimulus display, which remained visible until a response was detected or a timeout duration of 5000 ms was reached. Trials without a response were considered errors. Subjects were instructed to respond as accurately and quickly as possible. A 750 ms feedback display followed each response, presenting a black “+” to indicate a correct response and a black “X” to indicate an error.

The subjects completed 2 blocks of 4 practice trials and 8 blocks of 48 experimental trials. They were allowed to rest between blocks. At the beginning of each block, subjects were informed of which target (a right vs. left-handled cup) to search for. Each block contained all combinations of search set size and target presence and repeated eight times. Order of trials within each block was random.

### Statistical Analyses

Of our primary interest is to determine the presence or absence of specific effects of the independent variables. However, the traditional null-hypothesis significance tests (NHSTs) approach does not allow evidence in favor of the null (Wagenmakers, 2007; Cumming, 2013). Default Bayesian tests (Rouder and Morey, 2012) were employed to circumvent such issue of the NHSTs. Replacing *p*-values, Bayes factors (BFs) serve as measures of evidence for or against effects of interest. Briefly, BFs indicate relative likelihood of data favoring one hypothesis to the competing hypothesis. Therefore, if a BF favors the model that excludes a statistical effect than the model that includes it, then the data provide evidence against the effect. BFs are computed with the full model including an effect of interest in the numerator and the reduced model excluding the effect in the denominator. Following the nomenclature of Rouder and Morey (2012), these are labeled B_10_. A B_10_ value greater than 1.0 provides evidence for a statistical effect and a B_10_ value less than 1.0 provides evidence for the null. Interpretation of strength of evidence provided by BFs (anecdotal, substantial, strong, very strong, or decisive evidence) comes from Wetzels et al. (2011). To measure effect sizes, generalized eta-squared (Olejnik and Algina, 2003) is used.

## Results

Incorrect responses were excluded from analysis of RTs. For the analysis, linear regression equations were fit to the mean RTs by set size functions in each experimental condition for each subject (Wolfe, 1998). Slopes and intercepts of the linear regression equations were separately analyzed. Averaged across subjects and experimental conditions, linear functions accounted for 90.86% of the total variance in relationship between the mean RT and the set size. The slopes and intercepts were submitted to 2 × 2 × 2 × 2 analyses of variance (ANOVA) with Response Mapping and Stimulus Type as between-subject factors and Target Type (Left vs. Right handle) and Target Presence (Present vs. Absent) as within-subject factors. Preliminary analyses included Stimulus Type as a between-subject factor in the both analyses. In the analysis of slopes, the data gave anecdotal evidence that the type 1 stimuli produced larger slopes than the type 2 (*F*_(1,66)_ = 7.44, ${\text{η}}_{G}^{2}$ = 0.06, *B*_10_ = 2.67), but showed no evidence for an interaction of stimulus type with any other factors (all *B*_10_ < 0.54). In the analysis of intercepts, the data provided again anecdotal evidence for responses by the right hand faster than those by the left hand (*F*_(1,66)_ = 3.41, ${\text{η}}_{G}^{2}$ = 0.02, *B*_10_ = 1.96). To simplify exposition, we excluded Stimulus Type from the analyses of slopes and intercepts.

### Intercepts

**Figure 2:**
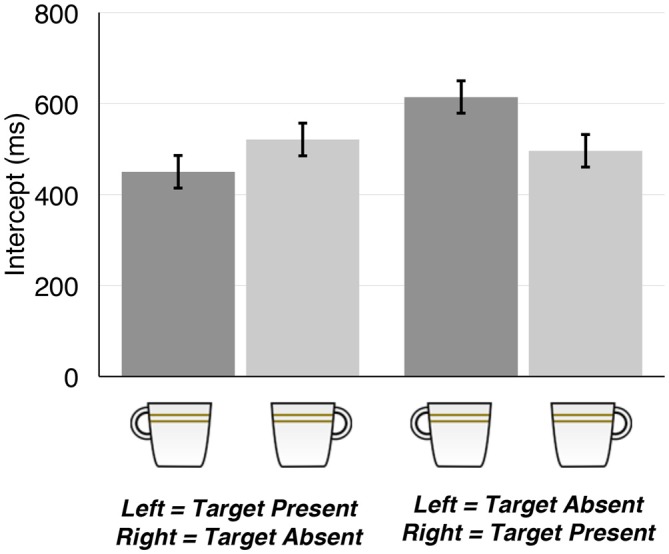
**Mean intercepts as a function of Target Type and Response Mapping.** Error bars represent 95% within-subject confidence intervals (Loftus and Masson, 1994) based on the main effect of Target Type.

Figure 2 presents mean intercepts as a function of stimulus type and response mapping. Target-present trials produced decisively larger intercepts than the target-absent trials (*F*_(1,66)_ = 13.00, ${\text{η}}_{G}^{2}$ = 0.03, *B*_10_ = 845.00). The data provided strong evidence for a two-way interaction between Target Type and Response Mapping (*F*_(1,66)_ = 36.89, ${\text{η}}_{G}^{2}$ = 0.06, *B*_10_ = 9.56 × 10^5^), indicating an SRC effect (Figure 2). *Post hoc* Bayesian *t*-tests revealed that intercepts were decisively smaller when the target was left-handled than right-handled for subjects who reported the target presence with the left hand and the target absence with the right hand (*M* = 450.01 vs. 520.81 ms; paired-*t*_(69)_ = 3.94, *B*_10_ = 115.85), while this pattern was reversed for subjects with the revered response mapping (*M* = 614.28 vs. 496.18 ms; paired-*t*_(69)_ = 5.90, *B*_10_ = 24988). The data gave no substantial evidence for any other effects (0.19 < *B*_10_ < 1.35).

### Slopes

**Figure 3:**
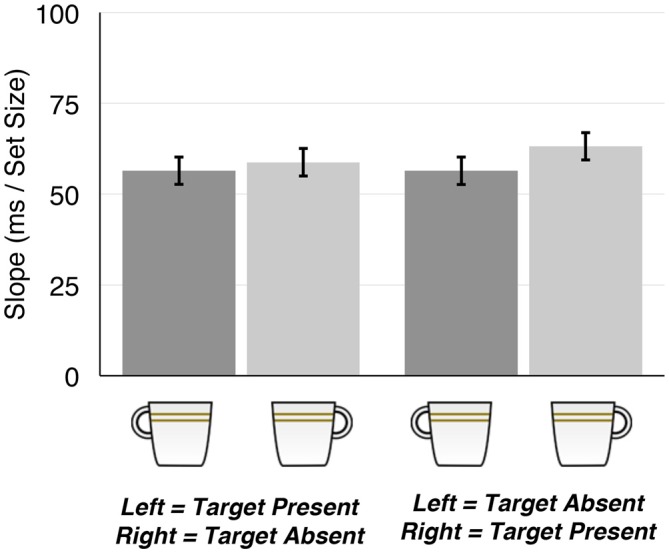
**Mean slopes as a function of Target Type and Response Mapping**.

As expected, RT slopes were decisively larger when the target was absent than when it was present (mean slope = 91.7 vs. 48.4, respectively; *F*_(1,68)_ = 116.26, ${\text{η}}_{G}^{2}$ = 0.29, *B*_10_ = 1.58 × 10^35^). However, data gave no strong evidence either for or against a main effect of target orientation (left-handled vs. and the right-handled; Figure 3; *F*_(1,68)_ = 7.75, ${\text{η}}_{G}^{2}$ = 0.004, *B*_10_ = 0.60), and gave substantial evidence against any other effects (0.26 < *B*_10_ < 0.33).

### Error Rates

Arcsin transformed error rates were submitted to an omnibus ANOVA with Response Mapping and Stimulus Type as between-subject factors and Target Type, Target Presence, and Set Size as within-subject factors. The data gave substantial evidence against the effect of Target Type and no evidence for interaction effects involving Target Type. Therefore, the data were then submitted to a 2 × 2 × 2 × 3 ANOVA without Stimulus Type as a factor. Subjects made decisively more errors in the target-present than the target-absent trials (0.06 vs. 0.02; *F*_(1,204)_ = 114.15, ${\text{η}}_{G}^{2}$ = 0.10, *B*_10_ = 1.83 × 10^28^). Error rates progressively and decisively increased as the number of search items increased (*F*_(2,204)_ = 12.14, ${\text{η}}_{G}^{2}$ = 0.05, *B*_10_ = 1.01 × 10^13^), and the rate of increase was decisively greater for the target-present than the target absent conditions (*F*_(2,204)_ = 26.26, ${\text{η}}_{G}^{2}$ = 0.05, *B*_10_ = 1.43 × 10^12^). Error rates data also showed an SRC effect: error rates were smaller when the direction of the handle and the button for a target-present response matched than mismatched (*F*_(1,204)_ = 18.46, ${\text{η}}_{G}^{2}$ = 0.01, *B*_10_ = 264.52). The data gave no substantial effect for any other effects (0.04 < *B*_10_ < 0.50). Thus, the data gave no evidence for speed-accuracy tradeoffs.

## Discussion

Two points summarize the present results. First, the affordance-offering stimuli produced SRC effect on intercepts of the search functions: Congruency between the direction of the handle and hand for the target-present response markedly reduced RTs intercepts. This result is consistent with previous reports that motor responses are faster when the handle of an object is aligned with the response hand than when it is misaligned (e.g., Riddoch et al., 1998; Tucker and Ellis, 1998; Creem and Proffitt, 2001; Vainio et al., 2007). Intercepts as described above indicate sensory processing and motor execution times. The data imply that the handled cup stimuli automatically triggered afforded motor responses more quickly than unafforded responses. Unexpectedly, the intercepts for the target-absent condition were smaller than those for the target-present condition. A standard serial self-terminating model of visual search (e.g., Sternberg, 1966; Wickens and McCarley, 2007) predicts similar sizes of intercepts. Because the target-present display contained one target and numerous distractor objects, subjects might have needed to resolve competing motor representations by inhibiting the incorrect response that is afforded by the distractors (e.g., Cisek and Kalaska, 2005; Cisek, 2007; Pastor-Bernier and Cisek, 2011). Further research is necessary for evaluating this hypothesis.

Second, search efficiency, measured by slopes of the search functions, was similar between the left-handled and the right-handled target conditions regardless of response mapping, suggesting that the congruency between the direction of the handle and hand for target-present response did not guide spatial attention preferentially to the target in the current paradigm. Recent works (Cho and Proctor, 2010; Pappas, 2014) suggest that affordance and the SRC effects are dissociable. According to Pappas (2014), the amount of object and depth information is necessary for providing the object’s affordance but not sufficient to trigger automatic responses. It follows that if it is the SRC that is responsible for automatic response activation, then search efficiency should not differ between the left- and the right-oriented targets because the SRC effect should only arise at the level of post-search process including response selection and execution. In fact, the present results concord with the prediction. Furthermore, preliminary data we have collected using the inverted cup stimuli support this view: with the inverted cups, which presumably provides less affordance than the upright cups, the SRC effect was observed (Target Type × Response Mapping interaction, *F*_(1,18)_ = 21.33, ${\text{η}}_{G}^{2}$ = 0.04, *B*_10_ = 18.11) while the data gave substantial evidence against the effect of Target Type on slopes (*B*_10_ = 0.23). Taken together, the current cup stimuli could have affected post-search processes but not attentional prioritization of the afforded target among distractors.

Graspable objects can automatically attract visual attention to the location of the objects (e.g., Handy and Tipper, 2007) and action preparation can also affect allocation of visual attention (e.g., Wykowska et al., 2009; Buttaccio and Hahn, 2011), processes essential for efficient visuomotor processing. The current results imply that object graspability have little influence on attentional prioritization in the visual search task, while speeding post-search response execution due to the SRC.

## Author Contributions

YY and AA designed research. YY conducted research and analyzed data. YY and YY wrote the article.

## Conflict of Interest Statement

The authors declare that the research was conducted in the absence of any commercial or financial relationships that could be construed as a potential conflict of interest.
